# Genotoxicity Assessment and Protective Effect of *Anogeissus leiocarpus* Roots against Cyclophosphamide-Induced DNA Damage In Vivo

**DOI:** 10.1155/2021/8020240

**Published:** 2021-11-08

**Authors:** Aku Enam Motto, Povi Lawson-Evi, Aboudoulatif Diallo, Kwashie Eklu-Gadegbeku

**Affiliations:** ^1^Laboratory of Physiology/Pharmacology, Unit of Pathophysiology, Bioactive Substances and Safety, Faculty of Sciences, University of Lome, B.P. 1515, Lome, Togo; ^2^Department of Toxicology, Faculty of Health Sciences, University of Lome, B.P. 1515, Lome, Togo

## Abstract

**Background:**

Belonging to the family of Combretaceae, the roots of *Anogeissus leiocarpus* are traditionally used to treat diabetes, wounds, infections, pain, and gastrointestinal diseases. To our knowledge, no genotoxicity assessment of the plant was reported. Hence, this study was designed to evaluate the potential genotoxic and protective effects of extract of *Anogeissus leiocarpus* roots using the micronucleus test on mice bone marrow cells in vivo.

**Methods:**

Three different concentrations (250, 500, and 1000 mg·kg^−1^) of hydroalcoholic extract of roots of *A*. *leiocarpus* were administered daily for 7 days per os to mice, and the genotoxicity was induced by the administration ip of cyclophosphamide. Genotoxicity and cytotoxicity were evaluated by counting, respectively, the number of micronucleated polychromatic erythrocytes and polychromatic erythrocytes to total erythrocytes in the bone marrow of mice.

**Results:**

The administration of *A*. *leiocarpus* did neither increase the ratio of the polychromatic erythrocyte (PCE) nor the frequency of micronucleated PCE (MNPCE) significantly in the bone marrow cells of the mice, compared to the vehicle control animals. However, a significant increase in the incidence of MNPCE in the bone marrow cell of the cyclophosphamide-treated mice was found. Moreover, in the groups treated with the total extract of *A*. *leiocarpus* at different doses plus cyclophosphamide, there was a significant decrease (*p* < 0.0001) in MNPCEs compared to the positive controls, in a dose-dependent manner.

**Conclusion:**

This first finding reports that the extract of *A*. *leiocarpus* was neither genotoxic nor cytotoxic. However, it shows a protective effect against the genotoxicity and cytotoxicity induced by cyclophosphamide.

## 1. Introduction

Cyclophosphamide is an alkylating chemotherapeutic drug extensively used to treat many types of cancers including lymphoma, leukemia, breast, ovarian, and lung carcinomas [1]. However, its clinical use can lead to various organ toxicities [2, 3]. Cyclophosphamide has been reported to induce dominant lethal mutation, micronuclei, DNA damage, and generation of free radicals or reactive oxygen species (ROS), chromosomal aberrations, sister chromatid exchanges, and gene mutations. This can lead to a multitude of pathological conditions as mutagenicity, carcinogenicity, teratogenicity, myelosuppression, immunosuppression, reproductive toxicity, cardiac toxicity, lung toxicity, and urotoxicity [4–8].

The clinical efficacy of cyclophosphamide is restricted due to its undesired toxicities in normal cells. Therefore, it is important to prevent normal cell DNA damage induced by cyclophosphamide [9]. Plants may offer new alternatives to these limited therapeutic options. Therefore, it has been an increased scientific interest in the study of materials from a plant source as an anticancer compound [10, 11]. Many studies in the literature have reported the expressive anticarcinogenic and antimutagenic activities of plants that contained phenolic compounds, flavonoids, and tannins due to their antioxidant properties [12–14].

*Anogeissus leiocarpus*, a plant of the Combretaceae family, is widely used in traditional medicine in Togo to treat various pathologies wounds, infections, pain, gastrointestinal diseases, and diabetes [15, 16]. The phytochemical constituents of *Anogeissus leiocarpus* were found to be polyphenolic compounds, flavonoids, and triterpenes [17, 18].

Our previous works had reported the antihyperglycemic, lipid-lowering, and antioxidant activities of the total extract and fractions of roots of *Anogeissus leiocarpus* [19, 20]. Administered at daily repeated doses of 500 mg·kg^−1^ and 1000 mg·kg^−1^, the hydroalcoholic extract of *A*. *leiocarpus* is found to be nontoxic in rats following either a single dose or daily repeated doses during 28 days in vivo [21].

Despite the large use of roots of *Anogeissus leiocarpus*, no report on its genotoxicity assessment is available in the literature. Thus, based on the pharmacological activity of this plant, especially its strong antioxidant properties, this current study was investigated to evaluate the potential genotoxic as well as the protective effects of hydroalcoholic extract of *Anogeissus leiocarpus* roots using the micronucleus test on mice bone marrow cells in vivo.

## 2. Materials and Methods

### 2.1. Chemical and Reagents

Cyclophosphamide monohydrate was purchased from Sigma-Aldrich (St. Louis, MO). Other commercial reagents (May Grunwald, formaldehyde, and Giemsa) were obtained from Biolabo S.A. (Paris, France)

### 2.2. Animals

Male ICR mice (30 ± 5 g) were kept in standard environmental conditions (temperature 24-25°C, relative humidity, and a 12 t/12 h light-dark cycle) and fed with standard rat diet and water ad libitum. Female mice were excluded from the study because of their cyclic hormonal variations. This study has been approved by the Ethics Committee of the University of Lomé, a branch of the National Ethics Committee for control and supervision of experiments on animals (NSBM/UL/14/NS0004).

### 2.3. Plant Material

Roots of *Anogeissus leiocarpus* were harvested in Tsévié, Zio (Togo). A voucher specimen was identified and deposited in the herbarium of the Laboratory of Botany and Plant Ecology under the number TOGO 15483. Roots of *Anogeissus leiocarpus* were cleaned out with water, cut into small pieces, dried at the Animal Physiology laboratory at 22°C, and then reduced into powder using the Mill Thomas Scientific^TM^.

### 2.4. Plant Extraction

About 400 g of roots of *Anogeissus leiocarpus* were extracted in water/ethanol (5 : 5) for 72 hours. The crude extract was filtered on Whatman paper and evaporated in a vacuum at 45°C using a rotary evaporator (IKA^®^ RV 10 digital). The yield of the dry extract was 5.68% and was stored at 4°C [18].

### 2.5. Reported Phytochemical Analysis of Roots of *Anogeissus leiocarpus*

A phytochemical study performed on *Anogeissus leiocarpus* revealed the isolation of polyphenolic compounds such as 3,3,4-tri-O-methylflavellagic acid, 3,3,4-tri-O-methylflavellagic acid-4-D-glucoside, gentisic acid, protocatechic acid, chebulagic acid, chebulinic acid, and ellagic acid. Flavogallonic acid bislactone, castalagin, and ellagic acid were isolated from the bark. Eight flavonoids such as 4H-1-benzopyran-4-one, 7-[(6-deoxy-*α*-L-mannopyranosyl)oxy]-5-hydroxy-2-(4-hydroxy-3-methoxyphenyl), catechin, quercetin, isoquercetin, rutin, vitexin, kaempferol, and procyanidin B2 were isolated from the leaves of the plant. Five triterpenes and triterpene glycosides were isolated, namely, sericoside; its related aglyconesericic acid, rachelosperoside; its related aglyconerachelosperogenin, and arjungenin [17]. Our previous preliminary work also confirmed an importance of the amount of phenolic compounds, tannins, flavonoids, and polysaccharides [20].

### 2.6. Genotoxicity and Antigenotoxicity Assessment by the Micronucleus Test of Roots of *Anogeissus leiocarpus* in Mice Bone Marrow

OECD 474 test guidelines [22] with slight modifications for the mammalian erythrocyte micronucleus test states was used for this test on mice, divided into 8 groups of 5 animals. Three different concentrations of *A*. *leiocarpus* have been tested for potential genotoxic effects and cytotoxic activities in vivo in bone marrow cells of mice. The choice of doses of 250, 500, and 1000 mg·kg^−1^ was based on the therapeutic dose of *A*. *leiocarpus* that has been determined in our previous studies [19, 20].

#### 2.6.1. Experimental Design

To evaluate the possible toxic effect of the plant, the animals were divided into 5 groups as follows: group 1 received distilled water and served as a negative control. Group 2, a positive control received a dose of 100 mg·kg^−1^ of cyclophosphamide I.P. Three doses of 250 mg kg^−1^ bw, 500 mg kg^−1^ bw, and 1000 mg·kg^−1^ bw of hydroalcoholic extract of *Anogeissus leiocarpus* dissolved in distilled water had been administered daily for 7 days per os, respectively, to mice of groups 3, 4, and 5.

In order to detect the protective effect of *Anogeissus leiocarpus*, three doses of 250 mg·kg^−1^ bw, 500 mg·kg^−1^ bw, and 1000 mg·kg^−1^ bw of *Anogeissus leiocarpus* had been administered daily during 7 0 days per os, respectively, to mice of groups 6, 7, and 8. After 7 days of daily pretreatment, mice of groups 6, 7, and 8 received cyclophosphamide intraperitoneal (i.p.) at a dose of 100 mg·kg^−1^ bw.

Thirty hours after injection, the animals were anesthetized using light diethyl ether and sacrificed by cervical dislocation. All mice were observed daily for signs of toxicity during the treatment period. The bodyweight of each mouse was measured twice: before administration of the extracts and before sacrifice.

#### 2.6.2. Preparation of Slides

According to the method used by [23], with slight modifications, the femur was immediately removed. The epiphyses were cut, and the bone marrow was flushed out using 1 mL saline solution (NaCl 0.9%). The supernatant was discarded after 7 minutes of centrifugation. Formaldehyde (4%) in distilled water was added to preserve the cytoplasm. The cell suspension was softly smeared on the surface of the slide. Dried slides were fixed in May Grunwald for 2 min and stained with 5% Giemsa solution for 30 minutes. Stained slides were analyzed under the light microscope at the 1000 × magnification. Slides were observed using a binocular microscope (zigzag orientation) type “Olympus” and marking ϹЄ with a 100 × immersion objective. Images were taken directly on the laptop connected to the ocular camera type 049002-VGA (Germany) ϹЄ integrated into the microscope.

#### 2.6.3. Micronucleus and Erythrocyte Counting

A total of 5000 polychromatic erythrocytes (PCEs) per animal were scored for the incidence of micronucleated immature erythrocytes. The frequency of micronucleated polychromatic erythrocytes (MNPCEs) was expressed as a percentage. Besides, the number of PCE was counted in 1000 total number of erythrocytes (TE = PCE + NCE), and a ratio between PCE and TE represented the frequency of PCE.

  % MNPCE = ((number of MNPCE)/(TE)) × 100
  % PCE = ((number of PCEs)/(TE)) × 100
  MNPCE = micronucleus in polychromatic erythrocyte
  PCE = polychromatic erythrocyte
  NCE = normochromatic erythrocyte

### 2.7. Statistical Analysis

Data were expressed as mean ± SEM (standard error of the mean) using the GraphPad Prism 7 software. Statistical differences between groups were determined by ANOVA followed by the Bonferroni test and considered significant for *p* < 0.05.

## 3. Results

### 3.1. Evaluation of Genotoxic, Antigenotoxic, or Cytotoxic Effects of *A*. *leiocarpus* In Vivo

#### 3.1.1. Effect of Total Extract on Bodyweight

During the treatment, no abnormalities were reported in behavior in the different groups compared to controls. The bodyweight of each mouse (at the beginning and end of treatment) is given in Table 1. No significant difference (*p* > 0.05) was found between the bodyweight gain of the treated groups compared to the negative controls.

#### 3.1.2. Genotoxic Assessment of the Total Extract of *Anogeissus leiocarpus*

The clastogenic/aneugenic potential of *Anogeissus leiocarpus* was investigated by the micronucleus test.

**Figure 1:**
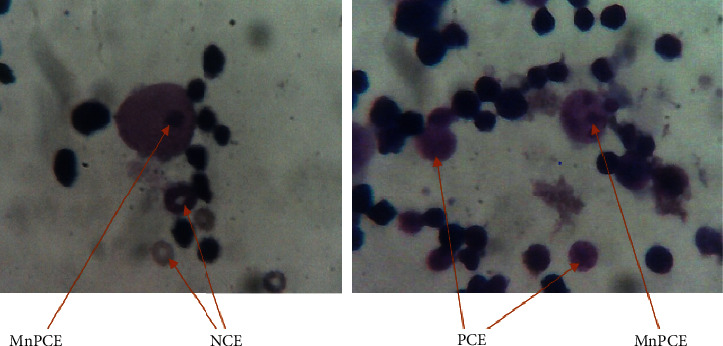
Representative images of micronucleus in polychromatic erythrocyte (MNPCE), polychromatic erythrocyte (PCE), and normochromatic erythrocyte (NCE). The bone marrow slides were stained with May Grünwald and Giemsa stain. Power oil immersion is 100*x*.

The administration of *A*. *leiocarpus* at the doses of 250, 500, and 1000 mg·kg^−1^ did increase neither the ratio of the polychromatic erythrocytes to total erythrocytes (PCEs%) in the bone marrow nor the frequency of micronucleated PCE (MNPCE) significantly in the bone marrow cells of the mice, compared to the vehicle control animals (Tables 2 and 3). However, a significant (*p* < 0.0001) increase up to 69% in the incidence of MNPCE in the bone marrow cell of the cyclophosphamide-treated mice was found (Figure 1).

The results showed no clastogenic/aneugenic effects of *Anogeissus leiocarpus*.

#### 3.1.3. Antigenotoxic Effect of Total Extract of *Anogeissus leiocarpus*

In the groups of animals which were pretreated with the total extract of *A*. *leiocarpus* (250, 500, and 1000 mg·kg^−1^) and then received cyclophosphamide, there was a significant reduction (*p* < 0.0001) in MNPCEs of 34, 44, and 62%, respectively, in a dose-dependent manner. This reduction of mutagenic effects was observed about the positive control group, which received cyclophosphamide without previous administration of the extract.

## 4. Discussion

The in vivo micronucleus test, first developed in mouse bone marrow erythrocytes, is one of the genotoxicity tests recommended by international regulatory agencies and government institutions for the evaluation of new substances [24]. Micronuclei (MN) are extranuclear bodies that contain damaged chromosome fragments and/or whole chromosomes that were not incorporated into the nucleus after cell division. Direct DNA damage or breakage, chromosomal aberrations, mitotic apparatus dysfunctions, and interference with DNA synthesis are the possible explanations of MN formation [25, 26]. When a bone marrow erythroblast turns into an immature erythrocyte (polychromatic erythrocyte, PCE), the main nucleus is expelled, and any micronuclei formed may remain in the cytoplasm. Detection of micronuclei in these cells is facilitated by the absence of the main nucleus.

In this study, genotoxicity, prevention of genotoxicity, and general cytotoxicity were evaluated in mice treated with the total extract of *Anogeissus leiocarpus*.

Prior to the micronucleus test, our study reported no abnormalities in the general behavior of the animals in the different treatment groups compared to the negative controls throughout the treatment. No difference was also observed between the bodyweight of the mice (at the beginning, and the end of treatment) and between the bodyweight gain of the treated groups compared to the controls.

The mutagenic potential of mutagen is estimated by the number of micronucleated polychromatic erythrocytes (MNPCE) in the bone marrow of rodents. An increase of the incidence of micronucleated polychromatic erythrocytes (MNPCE) in treated animals is indicative of induced chromosomal damage [22].

In our study, the administration of the total extract at different doses had not provoked any significant increase of the frequency of micronucleated PCE (MNPCE) in the bone marrow cells of the mice, compared to the vehicle control animals. This demonstrated a nongenotoxic effect of *Anogeissus leiocarpus*. As expected, cyclophosphamide (the positive control) induced a 69% increase in MNPCE in their bone marrow cell compared to the vehicle control animals.

Our results are consistent with other studies in the literature [24, 27].

Cyclophosphamide is a well-known anticancer agent with alkylating properties that induce gene mutations, chromosomal aberrations, micronuclei, and chromatid exchanges in somatic cells [25, 28, 29]. As a matter of fact, acrolein is one of the metabolites of cyclophosphamide that induces oxidative stress which leads to DNA damage of normal cells and toxicities to various target organs. Acrolein rapidly enters the cell and activates the intracellular reactive oxygen species and nitric oxide production, leading to peroxynitrite formation which ultimately damages the lipids, proteins, and DNA inside the cell [30].

In order to confirm the presence or absence of cytotoxicity, polychromatic erythrocytes (PCE) were determined in 5000 erythrocytes (PCE + NCE) by the ratio PCE/NCE. When bone marrow cells proliferation is affected by a toxic substance, a decrease in PCE/NCE ratio occurred reflecting bone marrow toxicity and cell depression [22].

The administration of the extract of *Anogeissus leiocarpus* showed no significant decrease in the PCE/PCE + NCE ratio in the treated groups compared to the negative control group. This suggests a noncytotoxic activity of the total extract of roots of *Anogeissus leiocarpus* on mouse bone marrow cells.

Moreover, in the groups of mice treated with the extract plus cyclophosphamide, the extract has shown a protective action against genotoxicity induced by cyclophosphamide in the bone marrow. Compared to positive controls, the number of MNPCEs decreased with the dose of extract administered. In fact, at the dose of 1000 mg·kg^−1^, the incidence of MNPCE was 1.39. 10-3 compared to 2.42. 10-3% at the dose of 500 mg·kg^−1^.

The major components of *Anogeissus leiocarpus* are polyphenolic compounds, flavonoids, and triterpenes [17, 18]. The antigenotoxic property of the plant may be related to its phytochemical components. In our earlier phytochemical and antioxidant studies, in vitro, the total extract had the ability to scavenge free radicals, reduce metal, and possessed strong total antioxidant activity. This strong antioxidant was due to phenolic compounds in the extract [20].

One of the important classes is flavonoids (quercetin, rutin) which exert their genoprotection by chelating the divalent cations, scavenging free radicals, and modulating the enzymes responsible for bioactivation of genotoxic agents and detoxification of their reactive metabolite [31].

Ellagic acid, a naturally occurring plant polyphenol, was evaluated for its antigenotoxicity and antioxidant efficacy against the cyclophosphamide-induced renal oxidative stress and genotoxicity in Swiss albino mice [32]. Thus, the presence of this polyphenol in our plant may be involved in preventing free radical-mediated cytotoxicity and lipid peroxidation.

Many studies in the literature exhibited a protective effect against the genotoxicity of triterpenoids and tannins in natural products [33, 34].

## 5. Conclusion

Hence, to our knowledge, this is the first published study that demonstrates that the hydroalcoholic extract of roots of *Anogeissus leiocarpus* is not genotoxic and not clastogenic at the concentrations used. In addition, the extract has the ability to prevent chromosomal damages caused by cyclophosphamide. Further studies are needed to investigate the molecular modes of action of the extract/compound (s) by Western blotting, real-time RT-PCR, fluorescence microscopy, and expression analyses for a better understanding and safe use of roots of *A*. *leiocarpus*.

## Figures and Tables

**Table 1:** Effect of total extract on bodyweight of mice.

Groups	Bodyweight (g)						
	1	2	3	4	5	*M* ± SEM	Gain of weight (g)
Negative control							
Beginning	30	29	34	30	27	30 ± 1.14	**2.0** ± **0.5**
End	32	33	35	31	29	32.0 ± 1.0	
Positive control							
Beginning	32	25	30	28	24	27.8 ± 1.5	**3.0** ± **0.4**
End	36	29	33	30	26	30.8 ± 1.7	
T.E 250							
Beginning	32	29	22	22	25	26.0 ± 1.9	**2.6** ± **0.5**
End	35	30	24	26	28	28.6 ± 1.8	
T.E 500							
Beginning	28	32	32	29	31	30.4 ± 0.8	**2.0** ± **0.4**
End	29	35	33	32	33	32.4 ± 1.0	
T.E 1000							
Beginning	25	30	35	32	34	31.2 ± 1.7	**1.4** ± **0.2**
End	27	31	36	34	35	32.6 ± 1.6	
T.E 250 + CP							
Beginning	32	28	30	25	30	29.0 ± 1.1	**2.2** ± **0.3**
End	33	30	32	27	34	31.2 ± 1.2	
T.E 500 + CP							
Beginning	27	30	31	29	28	29.0 ± 0.7	**2.4** ± **0.5**
End	30	32	35	31	29	31.4 ± 1.0	
T.E 1000 + CP							
Beginning	28	30	30	28	25	28.2 ± 0.9	**2.0** ± **0.4**
End	29	33	31	30	28	30.2 ± 0.8	

Negative control, treated with distillated water; positive control, treated only with cyclophosphamide at 100 mg·kg^−1^; T.E 250, 500, and 1000, treated, respectively, with the total extract at 250, 500, and 1000 mg·kg^−1^; T.E 250 + C, 500 + C, and 1000 + C, pretreated, respectively, with the total extract at 250, 500, and 1000 mg·kg^−1^ and received cyclophosphamide at 100 mg·kg^−1^. Bodyweight was recorded at the beginning of the experimentation and at the end. Bodyweight gain = ending bodyweight–bodyweight at the beginning.

**Table 2:** Genotoxic assessment of the total extract of *Anogeissus leiocarpus*.

Mice	Negative control		T.E 250		T.E 500		T.E 1000		Positive control	
	MNPCEs/5000 PCEs	PCEs%	MNPCEs/5000 PCEs	PCEs%	MNPCEs/5000 PCEs	PCEs%	MNPCEs/5000 PCEs	PCEs%	MNPCEs/5000 PCEs	PCEs%
1	6.55	54.57	5	41.94	5.5	41.94	4.29	43.44	15.69	39.67
2	4.47	53.63	4	38.67	4.5	42.62	7	33.64	21	40
3	7.5	40.90	5.5	44.05	5	39.39	5.71	43.44	20	45.45
4	3.33	42.30	4.85	46.43	4	42.03	4,5	47.20	15	46.66
5	4.16	41.17	4.4	45.24	6	40.32	6	37.70	21	40
*M* ± SEM	**5.70** ± **0.78**	**46.52** ± **3.10**	**4.75** ± **0.25**	**43.26** ± **1.36**	**5** ± **0.35**	**41.26** ± **0.60**	**5.5** ± **0.5**	**41.08** ± **2.40**	**18.54** ± **1.32**^####^	**42.35** ± **1.52**

Negative control, treated with distillated water; positive control, treated only with cyclophosphamide at 100 mg·kg^−1^; T.E 250 + C, 500 + C, and 1000 + C, pretreated, respectively, with the total extract at 250, 500, and 1000 mg kg^−1^ and received cyclophosphamide at 100 mg·kg^−1^; MNPCE, micronucleus in polychromatic erythrocyte; PCE, polychromatic erythrocyte; NCE, normochromatic erythrocyte; ^####^*p* < 0.0001 (compared to normal); ^*∗∗∗∗*^*p* < 0.0001 (compared to controls). % PCE = ((number of PCEs)/(number of PCEs + number of NCE s)) × 100.

**Table 3:** Antigenotoxic effect of total extract of *Anogeissus leiocarpus*.

Mice	Negative control		Positive control		T.E 250 + CP		T.E 500 + CP		T.E1000 + CP	
	MNPCEs/5000 PCEs	PCEs%	MNPCEs/5000 PCEs	PCEs%	MNPCEs/5000 PCEs	PCEs%	MNPCEs/5000 PCEs	PCEs%	MNPCEs/5000 PCEs	PCEs%
1	6.55	54.57	15.69	39.67	12.5	44.28	11.25	41.66	8.33	40
2	4.47	53.63	21	40	12.5	45	11	40.62	6.25	43.33
3	7.5	40.90	20	45.45	10	42.5	9.33	36.36	6	37
4	3.33	42.30	15	46.66	13.33	46.66	10	42.5	7.25	41.66
5	4.16	41.17	21	40	12.3	44.2	10	45	6.95	42
*M* ± SEM	**5.70** ± **0.78**	**46.52** ± **3.10**	**18.54** ± **1.32**^####^	**42.35** ± **1.52**	**12.12** ± **0.56**^*∗∗∗∗*^	**44.53** ± **0.67**	**10.31** ± **0.35**^*∗∗∗∗*^	**41.23** ± **1.41**	**6.95** ± **0.41**^*∗∗∗∗*^	**40.80** ± **1.08**

Negative control, treated with distillated water; positive control, treated only with cyclophosphamide at 100 mg kg^−1^; T.E 250 + CP, 500 + CP, and 1000 + CP, treated, respectively, with the total extract at 250, 500, and 1000 mg kg^−1^ plus cyclophosphamide; MNPCE, micronucleus in polychromatic erythrocyte; PCE, polychromatic erythrocyte; NCE = normochromatic erythrocyte; ^####^*p* < 0.0001, compared with negative control; ^*∗∗∗∗*^*p* < 0.0001, compared with to the positive control). % PCE = ((number of PCEs)/(number of PCEs + number of NCE s)) × 100.

## Data Availability

The data used to support the findings of this study are available from the corresponding author upon request.
